# A protocol on the effects of interactive digital assistance on engagement and perceived quality of care of surgery patients and self-efficacy and workload of staff

**DOI:** 10.3389/fmed.2022.989808

**Published:** 2022-10-17

**Authors:** Nejc Plohl, Vojko Flis, Andrej Bergauer, Nina Kobilica, Tadej Kampič, Samo Horvat, Damjan Vidovič, Bojan Musil, Urška Smrke, Izidor Mlakar

**Affiliations:** ^1^Department of Psychology, Faculty of Arts, University of Maribor, Maribor, Slovenia; ^2^University Clinical Centre Maribor, Maribor, Slovenia; ^3^Faculty of Electrical Engineering and Computer Science, University of Maribor, Maribor, Slovenia

**Keywords:** digital assistance, socially assistive robots, surgery patients, healthcare employees, gamification

## Abstract

**Introduction:**

The workforce shortage in the healthcare context is a growing issue that exerts detrimental effects on employees (e.g., higher workload) and patients (e.g., suboptimal patient care). Since traditional approaches alone may not be enough to solve this problem, there is a need for complementary innovative digital health solutions, such as socially assistive robots. Hence, the proposed study aims to investigate the effects of gamified nursing education and physiotherapy delivered by a socially assistive robot on patient- (engagement, perceived quality of care) and employee-related outcomes (perceived self-efficacy, workload).

**Methods and analysis:**

Approximately 90 vascular and thoracic surgery patients will receive either standard care or standard care with additional robot interactions over the course of 3–5 days. Additionally, approximately 34 nursing and physiotherapeutic employees will fill out self-report questionnaires after weeks of not using a social robot and weeks of using a social robot. The main hypotheses will be tested with mixed-design analyses of variance and paired-samples *t*-tests.

**Discussion:**

While the proposed study has some limitations, the results will provide high-quality and comprehensive evidence on the effectiveness of socially assistive robots in healthcare.

**Ethics and dissemination:**

The study was approved by the Medical Ethics Commission of the University Medical Center and registered in the ISRCTN registry (ISRCTN96689284). The study findings will be summarized in international peer-reviewed scientific journals and meetings and communicated to relevant stakeholders.

## Introduction

The workforce shortage is one of the main challenges faced by the current healthcare system; for example, only in the United Kingdom, the National Health Service reported a workforce shortage of around 100,000 employees in 2019 (1). These numbers, particularly the demand for nurses, are projected to increase due to various reasons, most notably due to population aging (2). The National Health Service estimates that shortages could grow up to 200,000 by the middle of the decade and at least to 250,000 by 2030 (1). Naturally, workforce shortage, together with increasing demands and having to take care of patients who are sicker than in the past (due to reduced patient length of stay), leads to a higher workload of the existing staff (2). On the provider side, this creates a vicious circle; increased workload negatively affects job satisfaction, increases burnout and turnover, and thus additionally contributes to the shortage of employees (3, 4). On the end-user side, employees’ higher workload is associated with suboptimal patient care and may lead to reduced patient satisfaction (4, 5). As a higher workload can lead to omissions of essential nursing care (6), nursing tasks that are generally not a priority, but are important nonetheless, can suffer even more (7). Such overlooked tasks may include educating patients, increasing patients’ health literacy, engaging in non-critical communication, and empowering patients to take a more proactive role in decision-making.

Considering that achieving active patient engagement and self-management is one of the leading healthcare goals of the twenty-first century (8, 9) and that the already overworked staff cannot always sufficiently fulfill this goal, there is a need for innovative solutions that may complement the existing approaches (for example, hiring additional nurses may only help to a certain extent, as the nursing schools are not able to keep up with the increasing educational demand (10)). The gap may be reduced with digital health approaches, which refer to the use of information and communications technologies in medicine and other health professions. Digital health can involve both hardware and software solutions and may serve different functions, including improving the quality of care, providing more personalized healthcare to patients, and improving the overall patient and staff experience (11). While “digital health” is generally a very broad term, socially assistive robots, i.e., robots that assist human users through social interaction, seem to be a particularly promising technology-based solution in this context (12). First, they were designed to provide education, feedback and support, coach patients through tasks, and serve the role of a companion (12–14). Second, many socially assistive robots are equipped with multimodal sensing and can thus collect a wide array of data that can be used for personalizing healthcare and individualized responding to patients’ needs and demands (15). Third, social robots can be developed in a way that facilitates patients’ motivation, learning, and confidence, for example, via integrating elements of gamification (16). Lastly, socially assistive robots can physically interact and work with humans while being immune to diseases such as COVID-19, demonstrating their suitability for situations that require physical distancing and isolation (17).

While hospitals and other healthcare institutions are increasingly adopting socially assistive robots (18, 19), research on their effects on patients and staff is scarce yet promising. In fact, the existing empirical studies suggest that socially assistive robots, if implemented properly, can positively impact patient engagement, satisfaction, and wellbeing (20–22). Furthermore, many healthcare workers perceive social robots as a tool that could alleviate their workload by supporting them to provide better care to older people [e.g., via assisting with medication, providing information, and fetching things (23)]. There is also tangible evidence that placing robots in healthcare institutions can help reduce staff workload (20, 24, 25). However, the generalizability of these studies is somewhat limited as they often employ rather rudimental methodological approaches and relatively small and specific samples, such as the elderly with dementia and children with neurodevelopmental disorders (12, 26–28). Moreover, to our best knowledge, no study has specifically tested the effects of using a socially assistive robot capable of delivering gamified nursing education and general/respiratory physiotherapy.

Hence, the present study, carried out within the H2020 HosmartAI project, aims to investigate the effects of a socially assistive robot Pepper (29)—that will be deployed on vascular and thoracic surgery wards and, at this stage, be partially operated and supervised by humans—on a wide array of patient- and employee-related outcomes.

As socially assistive robots can provide education without the usual restraints imposed upon human employees (e.g., the robot can effortlessly repeat the same instructions multiple times) and in a gamified manner that may facilitate motivation and learning, we expect positive effects of interactive digital assistance on patient-related outcomes. More specifically, we hypothesize that patients will exhibit higher levels of patient engagement (H1) and perceived quality of medical care (H2) after being subjected to standard care with additional gamified nursing education and general/respiratory physiotherapy (delivered by a socially assistive robot) compared to levels at the beginning of the study. Furthermore, we hypothesize that patients exposed to standard care with additional gamified nursing education and general/respiratory physiotherapy (delivered by a socially assistive robot) will experience larger improvement of patient engagement (H3) and perceived quality of medical care (H4) compared to patients exposed solely to standard care (human interaction only). Additionally, due to socially assistive robots’ potential to be involved in patient care and help with some of the work tasks, we hypothesize that employees will exhibit higher self-efficacy (H5) and experience lower workload (H6) during time periods when a socially assistive robot is deployed compared to time periods when a socially assistive robot is not deployed.

In addition to our primary hypotheses, we aim to determine the percentage of non-urgent communication taken up by the social robot (RQ1), the amount of employees’ time saved by such robot-human interactions (RQ2), and the extra time of interaction provided to patients by using a socially assistive robot (RQ3). We are also interested in how these variables (e.g., the number/percentage of AI interactions) correlate with both patient- and staff-related outcomes (RQ4). Lastly, we are also interested in patients’ user experience concerning the use of a socially assistive robot (RQ5). Answering these research questions and testing the hypotheses stated above will generate a novel and comprehensive insight into the effectiveness of using socially assistive robots in healthcare.

## Methods and analysis

### Socially assistive robot Pepper

The Pepper robot, developed by SoftBank Robotics, is a 120 cm tall social humanoid robot optimized for human interaction and engaging with people through conversation and a touch screen. It is capable of natural movement, navigation, speech recognition, and dialogue, and is equipped with perception modules and various sensors for multimodal interactions (e.g., microphones, infrared sensors, cameras, and sonars). These functions allow for smooth interaction that does not require any specific training. The Pepper robot also has several safety mechanisms, such as bumper sensors, that prevent it from physically harming participants (29). For the purposes of the proposed study (and other studies within the H2020 project HosmartAI), the robot was taught to understand and express gestures, facial expressions, and speech in the Slovenian cultural context and language. It was also taught to perform exercises and scenarios that are part of the intervention (see section “Study design”).

The robot was first extensively tested in a laboratory setting. After this stage, we introduced the robot to employees in both participating departments. The robot was then extensively pilot tested on-site over the period of 3 months, with significant input from the clinical staff.

Despite extensive pilot testing, Pepper will be constantly supervised throughout the study by at least one researcher, enabling us to detect failures immediately and make quick repairs if needed. In the case of more severe malfunctions, the robot will be temporarily replaced with a backup unit. Moreover, in the rare event of the second unit also malfunctioning, the study will be temporarily put on hold and participants from the interrupted iteration will be excluded from analyses. The study will continue when at least one robot is working properly again.

### Study design

The proposed study employs a between-within interaction design with multiple iterations. Patients and employees participating in the study will be recruited in a University Clinical Centre Maribor, Slovenia. The study will be carried out in 1-week intervals, followed by a 1-week washout period between each iteration. Overall, the study will be conducted over 2 years or less if the desired sample size is reached sooner.

**FIGURE 1:**
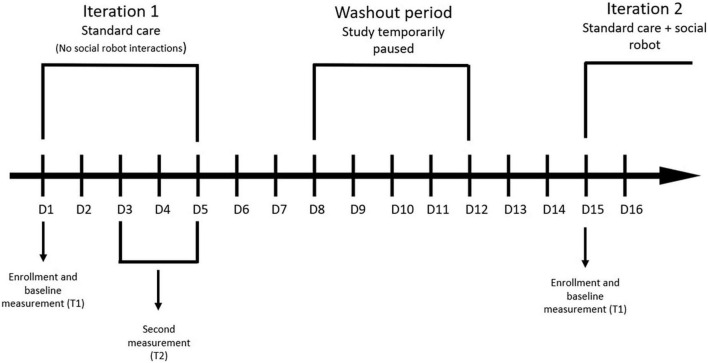
Study design: patients.

Patients admitted to vascular and thoracic surgery wards for an elective (non-emergency) procedure will be screened for eligibility regarding the inclusion and exclusion criteria. Eligible participants will then be informed about the study characteristics and asked to fill out an informed consent form. Those who will consent to participate in the study will participate for 3–5 days. Depending on the week (see Figure 1), participants will be allocated to the standard care group (i.e., a control group that will not interact with a socially assistive robot) or standard care group with additional robot interactions. Participants from both groups will first be informed about the study and fill out the baseline questionnaires (demographic data, engagement, and perceived quality of care). Similarly, participants from both groups will be subjected to standard nursing education and general/respiratory physiotherapy delivered by nursing and physiotherapy staff. However, the patients allocated to the standard care group with additional robot interactions will additionally be subjected to nursing education and general/respiratory physiotherapy delivered by a socially assistive robot. Moreover, they will be able to call the robot for additional assistance (not related to nursing education and physiotherapy) during the whole study period. At the end of their participation, patients from both groups will be asked to fill out the questionnaires (engagement, perceived quality of care) for the second time. Patients from both groups will also be asked to rate the education and physiotherapy they received during the study period. Moreover, patients that will interact with a socially assistive robot, will also fill out the User Experience Questionnaire (30) at the end their participation.

The study will be conducted in several 1-week iterations with a washout period between them (to provide enough time for new patients to be admitted). Specifically, patients will receive standard care in week 1, followed by a washout period in week 2. Patients in week 3 will then receive standard care together with robot interactions, followed by a washout period in week 4. In week 5, patients will again receive standard care. The study procedure (for patients) is outlined in Figure 1. It is worth noting that all additional variables (e.g., number of interactions with a robot) will be collected continuously during each relevant iteration. Additionally, the instructions of all self-report questionnaires will be adapted to reflect the chosen time periods and tasks. Throughout the study, patients will be observed for signs of high distress and excluded from the study if needed.

In terms of intervention content, the nursing education will contain critical information on wound care, home care considerations, and daily care explanations. On the other hand, general and respiratory physiotherapy will consist of selected instructions and exercises that can be delivered both by a human and a robot. The AI-delivered nursing education and physiotherapy instructions will employ elements of gamification.

**FIGURE 2:**
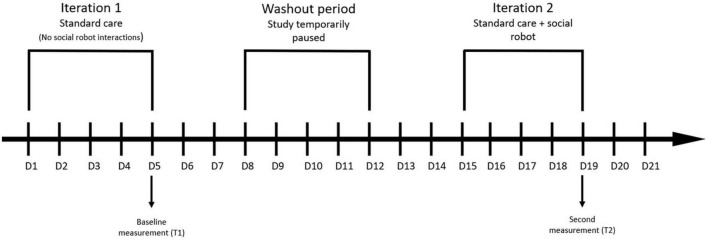
Study design: staff. This figure depicts only the first two measurements. The same protocol will be carried out at the 1-year mark and right before the end of the study (approximately 2 years after the beginning).

Similarly to patients, nursing and physiotherapy staff working on vascular and thoracic surgery wards will first be screened for eligibility regarding the inclusion and exclusion criteria. Eligible participants will then receive a brief description of the study and an informed consent form. Those who will consent to participate in the study will fill out the questionnaires twice, over a 3-week period. They will first fill out the questionnaires after a week of standard care (end of week 1: baseline self-efficacy and workload) and then again at the end of the first iteration of using a socially assistive robot (end of week 3: self-efficacy and workload after the intervention). To increase the robustness of results, the same protocol will be carried out thrice during the course of the study; at the initial deployment of a robot, after 1 year (T3 and T4), and right before the end of the study (T5 and T6). This will also enable us to investigate whether the difference between self-efficacy/workload at the time of robot use and non-use changes over time (once employees become more accustomed to the robot). The study procedure (for employees) is outlined in Figure 2. Again, the instructions of all self-report questionnaires will be adapted to relevant time periods and tasks.

### Participants and recruitment

The proposed study intends to recruit two distinct samples: patients and staff. The patient sample will be composed of patients admitted to vascular surgery and thoracic surgery wards for an elective (non-emergency) procedure. Beyond the type of disease for patients, patients need to be aged 18 years or above and willing to participate in the study. On the other hand, exclusion criteria include emergency patients, patients already enrolled in other studies, patients with dementia, special needs or appointed guardians, and patients allocated to an intensive step-down unit and/or regimen.

The staff sample will be composed of nursing and physiotherapy staff working on either the vascular surgery or the thoracic surgery ward. Again, only employees aged 18 years or above, who have signed a consent form, will be invited to participate in the study.

To minimize background noise while ensuring that all patients also receive standard care, the study will employ a between-within interaction design, whereby patients will be allocated either to the (1) standard care group or (2) standard care with additional robot interactions. We performed the sample size calculation in the G*Power software (31), choosing the ANOVA repeated measures with within-between interaction (2 groups, 2 measurements) as the statistical test, entering a small-to-medium effect size (*f* = 0.15), and choosing a conventional significance threshold and power (α = 0.05, 1-β = 0.80). Such calculations suggest that approximately 90 patients need to be recruited overall.

The nursing and physiotherapeutic staff will be studied with a simpler within-subjects design, whereby all consenting employees will fill out the questionnaires after a period of standard care and after the use of a socially assistive robot. As such, we performed the sample size calculation in the G*Power software, choosing the Paired-samples *t*-test as the statistical test, entering a medium effect size (*d* = 0.50), and choosing a conventional significance threshold and power (α = 0.05, 1-β = 0.80). Such calculations suggest that at least 34 nursing and physiotherapeutic employees must be recruited overall.

### Measures

Testing the primary hypotheses will require the measurement of several outcomes, namely patient engagement and perceived quality of medical care as well as self-efficacy and workload of staff. All questionnaires will be administered in Slovene. For questionnaires that have not yet been translated to this language and validated, we will use a standard procedure that involves translation and back-translation by qualified translators (32). Additionally, basic psychometric properties of the translated scales, such as internal reliability, will be tested prior to the main analyses.

Patient engagement will be measured with the Patient Health Engagement scale [PHE (33)], which consists of nine ordinal items. In the proposed study, we will convert the response format into a 5-point agreement scale ranging from “*completely disagree*” to “*completely agree*.” An example item is: “*Despite my illness, I know how to manage my life*.” The scale generally exhibits high correlations with concurrent measures, such as the Patient Activation Measure (34), and demonstrates solid reliability [test-retest and internal reliability (33)].

To measure the perceived quality of medical care, we will use the Perceived Quality of Medical Care scale [PQMC (35)]. The scale consists of six items (e.g., “*Medical care I have received was unsatisfactory/satisfactory*”), answered using a 7-point semantic differential scale. The scale is unidimensional and has previously exhibited excellent internal reliability [Cronbach α = 0.94 (36)].

The New General Self-Efficacy Scale [NGSE (37)] will be used to measure the extent to which employees believe they can achieve their goals. The NGSE is composed of eight items (e.g., “*I will be able to achieve most of the goals that I set for myself*”), which are answered on a 5-point agreement scale from “*strongly disagree*” to “*strongly agree*.” The overall score is then calculated by taking the average of all items. The scale is widely used in articles studying the adult population and generally exhibits high validity and reliability [Cronbach α = 0.86 (37)].

Lastly, employees’ workload will be measured with the Nasa Task Load Index [NASA TLX (38)], a very brief and widely used scale that assesses the perceived workload. The scale consists of six items (e.g., “*Temporal demand*”), answered on a 7-point scale ranging from “*very low*” to “*very high*.” The first three items will be slightly adapted to reflect the work in general instead of any one particular task. While each item alone provides useful and relevant information about different aspects of subjects’ experiences, the items can be combined into an overall score (39). Previous studies support the notion that the NASA TLX is a reliable and valid instrument also in the healthcare context [coefficient α = 0.72 (40)].

Answering the secondary objectives will require the assessment of additional outcomes, in particular, the percentage of non-urgent communication taken up by the social robot, time saved by human-robot interaction, and extra time of interaction provided to patients. Patients’ user experience and their ratings of each of the two types of nursing education (human/AI) and physiotherapy (human/AI) will also be assessed.

Most of these outcomes will not be measured with self-report questionnaires. During iterations with a socially assistive robot, the patients will be able to call for additional assistance and choose between human or AI assistance (by using one of two call devices). The number of calls will be recorded automatically, enabling us to calculate the ratio during analyses. Similarly, the number of actual interactions and the time spent for each encounter (with a human or AI) will also be recorded. Patients’ ratings of nursing education and physiotherapy delivered by a human/robot will be measured with a handful of self-construed items. It is worth noting here that both patients and staff will also be asked to fill out basic demographic questions.

Furthermore, patients will also fill out the User Experience Questionnaire [UEQ (30)]—the most widely adopted measure of user experience, which is already available in Slovene (41). It contains 26 items (e.g., “*unlikable/pleasing*”), which can form different subscores (i.e., perspicuity, efficiency, dependability, stimulation, and novelty) and the overall impression of the product. The subscales as well as the overall scale exhibit satisfactory internal consistency (for the overall score: coefficient α = 0.89).

### Data analysis

#### Preliminary analyses

We will use the IBM SPSS Statistics 28 program for statistical analysis. In the first step, we will clean the dataset and exclude all participants with more than 20% of missing values within any questionnaire. The remaining missing data will be analyzed to determine whether they are missing completely at random and, in case this is confirmed, replaced using the expectation-maximization algorithm. We will also perform basic psychometric analyses of the questionnaires, namely the analysis of reliability as internal consistency (coefficient α), and calculate the factor scores in accordance with the scoring instructions.

In the next step, we will perform basic descriptive analyses (means and standard deviations) and check the assumptions of the chosen statistical tests (such as the normality of the distribution assumption). This step will be followed by correlational analyses (to establish relationships between different variables) and inferential tests, whereby results with a *p*-value below 0.05 will be considered statistically significant.

#### Analyses of patient-related outcomes

The patient-related outcomes, specifically patient engagement (H1, H3) and perceived quality of care (H2, H4), will primarily be analyzed with two separate mixed-design (split-plot) analyses of variance (ANOVA), with group as the between-subjects factor (2 groups: standard care, standard care with robot interactions) and time as the within-subjects factor (2 levels: T1 and T2; see Figure 1). Significant results will be followed up with *post hoc* tests using the correction to adjust for multiple testing. All results will be accompanied by effect sizes.

#### Analyses of employee-related outcomes

The employee-related outcomes, namely self-efficacy (H5) and workload (H6), will be analyzed with two separate Paired-samples *t*-tests, with time as the within-subjects factor (2 levels: T1, T2; see Figure 2). All results will be accompanied by effect sizes.

#### Other analyses

The additional research questions will be answered descriptively (RQ1-RQ3, RQ5) or using the Pearson correlation coefficient (RQ4).

## Discussion

Despite the growing need for automated support and the constantly improving social robots, now capable of providing versatile services—such as education, social interaction, and entertainment—in a user-friendly manner, their large-scale implementation is progressing rather slowly (14, 17). This is disconcerting, as the existing studies suggest that their use is positively associated with the desired patient outcomes and can alleviate the workload of hospital staff (20–22, 24, 25). One of the plausible reasons for this gap is a relatively low number of systematic studies conducted in real-life settings, employing adequate sample sizes and investigating the effects on various outcomes. As put by Kvedar (42) when referring to digital health in general: “*One critical piece moving us along the curve is the accumulation of high-quality evidence, and there is no better way to curate evidence than through investigative inquiry*” (para. 6).

Our study is hence designed to extend evidence on gamified digital approaches and socially assistive robots in the context of healthcare. More specifically, this study’s main strength is that it will provide crucial data on the effects of gamified nursing education and general/respiratory physiotherapy delivered by a socially assistive robot on patients as well as staff. As such, it will provide some answers regarding the practical value of deploying social robots and their potential for facilitating engagement and perceived quality of care among patients. At the same time, it seeks to investigate whether social robots, as complementary devices to standard care, can reduce employees’ workload. To ensure a high enough quality of evidence provided by our study, we will conduct a rigorous study and use validated questionnaires to measure a wide variety of patient- and staff-related outcomes. Additionally, we will obtain a large enough sample of patients and employees. Throughout the process, we will follow the relevant recommendations and guidelines and pay attention to ethical considerations.

The proposed study is not without limitations. First, while our study will offer important insight into the effectiveness of interactive digital assistance, a randomized controlled trial with a larger sample size may be needed to further determine the effects of such intervention. Second, even though all surgery patients conforming with the inclusion criteria will be invited to participate in the study, it is plausible that only patients with more favorable attitudes toward technology will agree to participate in the study. This might lead to a sample that is generally younger and more educated (43, 44), possibly biasing the results on the effectiveness of social robot-delivered education and physiotherapy. Third, due to the specific characteristics of vascular and thoracic surgery patients (e.g., length of hospitalization), their exposure to the social robot will be somewhat short. Hence, the study will primarily focus on initial reactions to the socially assistive robot, whereas the trajectories of what happens over a more extended period (i.e., continued use), which can differ from initial reactions (45), will have to be explored in future studies. Lastly, in the present study, the social robot will be limited to a few specific services, physical locations, and one hospital, making it difficult to provide general and final conclusions on the effectiveness of social robots in hospitals.

## Ethics statement

This study was approved by the Medical Ethics Commission of the University Medical Center (UKC) Maribor on December 15th, 2021 (ID: UKC-MB-KME-76/21). The study findings will be published in international peer-reviewed scientific journals and presented at local and international scientific meetings. Summarized results will also be provided to healthcare professionals and other relevant stakeholders as well as the general public.

## Author contributions

IM and VF ensured funding for the study. NP, IM, and AB defined the study design. NP, AB, US, and IM wrote the original draft. IM, NP, US, and BM carried out background research. VF, AB, NK, TK, SH, and DV defined the primary and secondary outcomes. All authors contributed to the conceptualization of the study, reviewing and editing of the manuscript, read, and approved the final manuscript.

## References

[1] Gershlick B Charlesworth A. Health and social care workforce: Priorities for the new government. London: Health Foundation (2019).

[2] Carayon P Gurses AP. Nursing workload and patient safety — A human factors engineering perspective. In: Hughes RG editor. *Patient safety and quality: An evidence-based handbook for nurses.* (Rockville, MD: Agency for Healthcare Research and Quality) (2008). p. 203–16. 21328758

[3] Duffield C O’Brien-Pallas L. The causes and consequences of nursing shortages: A helicopter view of the research. *Aust Heal Rev.* (2003) 26:186–93. 10.1071/AH030186 15485390

[4] Pérez-Francisco DH Duarte-Clíments G Del Rosario-Melián JM Gómez-Salgado J Romero-Martín M Sánchez-Gómez MB. Influence of workload on primary care nurses’ health and burnout, patients’ safety, and quality of care: Integrative review. *Healthcare.* (2020) 8:12. 10.3390/healthcare8010012 31947761 PMC7151231

[5] Anderson FD Maloney JP Beard LW. A descriptive, correlational study of patient satisfaction, provider satisfaction, and provider workload at an army medical center. *Mil Med.* (1998) 163:90–4. 10.1093/milmed/163.2.90 9503899

[6] Griffiths P Recio-Saucedo A Dall’Ora C Briggs J Maruotti A Meredith P The association between nurse staffing and omissions in nursing care: A systematic review. *J Adv Nurs.* (2018) 74:1474–87. 10.1111/jan.13564 29517813 PMC6033178

[7] See MTA Chee S Rajaram R Kowitlawakul Y Liaw SY. Missed nursing care in patient education: A qualitative study of different levels of nurses’ perspectives. *J Nurs Manag.* (2020) 28:1960–7. 10.1111/jonm.12983 32096316

[8] Barello S Graffigna G Vegni E. Patient Engagement as an Emerging Challenge for Healthcare Services: Mapping the Literature. *Nurs Res Pract.* (2012) 2012:1–7. 10.1155/2012/905934 23213497 PMC3504449

[9] World Health Organizaton. *Patient engagement: Technical series on safer primary care.* Geneva: World Health Organization (2016).

[10] Kuehn BM. Global shortage of health workers, brain drain stress developing countries. *JAMA.* (2007) 298:1853–5. 10.1001/jama.298.16.1853 17954532

[11] Ronquillo Y Meyers A Korvek SJ. *Digital health.* Tampa, FL: StatPearls publishing (2021). 29262125

[12] Rabbitt SM Kazdin AE Scassellati B. Integrating socially assistive robotics into mental healthcare interventions: Applications and recommendations for expanded use. *Clin Psychol Rev.* (2015) 35:35–46. 10.1016/j.cpr.2014.07.001 25462112

[13] Feil-Seifer D Matarić MJ. Socially assistive robotics. *IEEE Robot Autom Mag.* (2011) 18:24–31. 10.1109/MRA.2010.940150

[14] Savage N. Robots rise to meet the challenge of caring for old people. *Nature.* (2022) 601:S8–10. 10.1038/d41586-022-00072-z 35046591

[15] Troccaz J Dagnino G Yang GZ. Frontiers of medical robotics: From concept to systems to clinical translation. *Annu Rev Biomed Eng.* (2019) 21:193–218. 10.1146/annurev-bioeng-060418-052502 30822100

[16] Feingold-Polak R Barzel O Levy-Tzedek S. A robot goes to rehab: A novel gamified system for long-term stroke rehabilitation using a socially assistive robot—methodology and usability testing. *J Neuroeng Rehabil.* (2021) 18:1–18. 10.1186/s12984-021-00915-2 34321035 PMC8316882

[17] Aymerich-Franch L. Why it is time to stop ostracizing social robots. *Nat Mach Intell.* (2020) 2:364. 10.1038/s42256-020-0202-5

[18] McGinn C Bourke E Murtagh A Donovan C Lynch P Cullinan MF Meet stevie: A socially assistive robot developed through application of a ‘design-thinking’ approach. *J Intell Robot Syst Theory Appl.* (2020) 98:39–58. 10.1007/s10846-019-01051-9

[19] Van Der Putte D Boumans R Neerincx M Rikkert MO De Mul M. A social robot for autonomous health data acquisition among hospitalized patients: An exploratory field study. *Proceedings of the ACM/IEEE international conference on human-robot interaction.* (Piscataway, NJ: IEEE) (2019). p. 658–9. 10.1109/HRI.2019.8673280

[20] Kachouie R Sedighadeli S Khosla R Chu MT. Socially assistive robots in elderly care: A mixed-method systematic literature review. *Int J Hum Comput Interact.* (2014) 30:369–93. 10.1080/10447318.2013.873278

[21] Kolstad M Yamaguchi N Babic A Nishihara Y. Integrating socially assistive robots into Japanese nursing care. *Proceedings of the 18th annual International conference on informatics, management, and technology in healthcare.* (Amsterdam: IOS Press) (2020). p. 183–6. 10.3233/SHTI200524 32604631

[22] Pu L Moyle W Jones C Todorovic M. The effectiveness of social robots for older adults: A systematic review and meta-analysis of randomized controlled studies. *Gerontologist.* (2019) 59:E37–51. 10.1093/geront/gny046 29897445

[23] Papadopoulos I Ali S Papadopoulos C Castro N Faulkes N Koulouglioti C. A qualitative exploration of care homes workers’ views and training needs in relation to the use of socially assistive humanoid robots in their workplace. *Int J Older People Nurs.* (2021) 17:e12432. 10.1111/opn.12432 34679219

[24] Khan ZH Siddique A Lee CW. Robotics utilization for healthcare digitization in global COVID-19 management. *Int J Environ Res Public Health.* (2020) 17:3819. 10.3390/ijerph17113819 32481547 PMC7312924

[25] Tietze M McBride S. *Robotics and the impact on nursing practice.* Maryland: American Nurses Association (2020).

[26] Aymerich-Franch L Ferrer I. Socially assistive robots’ deployment in healthcare settings: A global perspective. *ArXiv* [Preprint] (2021):1–28. 10.48550/arXiv.2110.07404 35895330

[27] Deutsch I Erel H Paz M Hoffman G Zuckerman O. Home robotic devices for older adults: Opportunities and concerns. *Comput Human Behav.* (2019) 98:122–33. 10.1016/j.chb.2019.04.002

[28] Kabacińska K Prescott TJ Robillard JM. Socially assistive robots as mental health interventions for children: A scoping review. *Int J Soc Robot.* (2021) 13:919–35. 10.1007/s12369-020-00679-0

[29] Softbank. *Pepper – The Humanoid and Programmable Robot.* Minato: Softbank (2022).

[30] Laugwitz B Held T Schrepp M. Construction and evaluation of a user experience questionnaire. *Proceedings of the symposium of the austrian HCI and usability engineering group.* Berlin: Springer (2008). p. 63–76. 10.1007/978-3-540-89350-9_6

[31] Faul F Erdfelder E Lang AG Buchner AG. * Power 3: A flexible statistical power analysis program for the social, behavioral, and biomedical sciences. *Behav Res Methods.* (2007) 39:175–91. 10.3758/BF03193146 17695343

[32] Bradley C. Translation of questionnaires for use in different languages and cultures. In: Bradley C editor. *Handbook of psychology and diabetes: A guide to psychological measurement in diabetes research and practice.* (London: Psychology Press) (1994). p. 43–56.

[33] Graffigna G Barello S Bonanomi A Lozza E. Measuring patient engagement: Development and psychometric properties of the patient health engagement (PHE) scale. *Front Psychol.* (2015) 6:1–11. 10.3389/fpsyg.2015.00274 25870566 PMC4376060

[34] Hibbard JH Mahoney ER Stockard J Tusler M. Development and testing of a short form of the patient activation measure. *Health Serv Res.* (2005) 40:1918–30. 10.1111/j.1475-6773.2005.00438.x 16336556 PMC1361231

[35] Richmond VP Smith RS Heisel AM McCroskey JC. The impact of communication apprehension and fear of talking with a physician and perceived medical outcomes. *Commun Res Rep.* (1998) 15:344–53. 10.1080/08824099809362133

[36] Richmond VP Heisel AM Smith RS McCroskey JC. The impact of communication apprehension and fear of talking with a physician on perceived medical outcomes. *Int J Phytoremediation.* (1998) 15:344–53. 10.1080/08824099809362133

[37] Chen G Gully SM Eden D. Validation of a new general self-efficacy scale. *Organ Res Methods.* (2001) 4:62–83. 10.1177/109442810141004

[38] Hart SG Staveland LE. Development of NASA-TLX (Task Load Index): Results of empirical and theoretical research. *Adv Psychol.* (1988) 52:139–83. 10.1016/S0166-4115(08)62386-9

[39] Hart SG. NASA-task load index (NASA-TLX); 20 years later. *Proc Hum Factors Ergon Soc.* (2006) 50:904–8. 10.1177/154193120605000909

[40] Hoonakker P Carayon P Gurses AP Brown R Khunlertkit A McGuire K Measuring workload of ICU nurses with a questionnaire survey: The NASA task load index (TLX). *IIE Trans Healthc Syst Eng.* (2011) 1:131–43. 10.1080/19488300.2011.609524 22773941 PMC3388621

[41] Debevc M Jazbec S Lapuh Bele J. *User experience questionnaire: Slovenian version.* (2016). Available online at: http://www.ueq-online.org/?slide=ueq-home (accessed September 27, 2022).

[42] Kvedar JC. Evidence for the effectiveness of digital health. *npj Digit Med.* (2020) 3:34. 10.1038/s41746-020-0231-9 32195369 PMC7064496

[43] Heerink M. Exploring the influence of age, gender, education and computer experience on robot acceptance by older adults. *HRI 2011 – proceedings of the 6th ACM/IEEE international conference on human-robot interaction.* (Piscataway, NJ: IEEE) (2011). p. 147–8. 10.1145/1957656.1957704

[44] Andtfolk M Nyholm L Eide H Rauhala A Fagerström L. Attitudes toward the use of humanoid robots in healthcare—a cross-sectional study. *AI Soc.* (2021):1–10. 10.1007/s00146-021-01271-4

[45] Wang T Fan L Zheng X Wang W Liang J An K The impact of gamification-induced users’ feelings on the continued use of mhealth apps: A structural equation model with the self-determination theory approach. J Med Internet Res. (2021) 23:1–15. 10.2196/24546 34387550 PMC8391751

